# AMD-Associated Genes Encoding Stress-Activated MAPK Pathway Constituents Are Identified by Interval-Based Enrichment Analysis

**DOI:** 10.1371/journal.pone.0071239

**Published:** 2013-08-05

**Authors:** John Paul SanGiovanni, Phil H. Lee

**Affiliations:** 1 Clinical Trials Branch, National Eye Institute, National Institutes of Health, Bethesda, Maryland, United States of America; 2 Analytic & Translational Genetics Unit, Center for Human Genetic Research, Massachusetts General Hospital, Harvard Medical School, Boston, Massachusetts, United States of America; Harvard Medical School, United States of America

## Abstract

**Purpose:**

To determine whether common DNA sequence variants within groups of genes encoding elements of stress-activated mitogen-activated protein kinase (MAPK) signaling pathways are, in aggregate, associated with advanced AMD (AAMD).

**Methods:**

We used meta-regression and exact testing methods to identify AAMD-associated SNPs in 1177 people with AAMD and 1024 AMD-free elderly peers from 3 large-scale genotyping projects on the molecular genetics of AMD. SNPs spanning independent AAMD-associated genomic intervals were examined with a multi-locus-testing method (INRICH) for enrichment within five sets of genes encoding constituents of stress-activated MAPK signaling cascades.

**Results:**

Four-of-five pathway gene sets showed enrichment with AAMD-associated SNPs; findings persisted after adjustment for multiple testing in two. Strongest enrichment signals (*P* = 0.006) existed in a c-Jun N-terminal kinase (JNK)/MAPK cascade (Science Signaling, STKE CMP_10827). In this pathway, seven independent AAMD-associated regions were resident in 6 of 25 genes examined. These included sequence variants in: 1) three MAP kinase kinase kinases (MAP3K4, MAP3K5, MAP3K9) that phosphorylate and activate the MAP kinase kinases MAP2K4 and MAP2K7 (molecules that phosphorylate threonine and tyrosine residues within the activation loop of JNK); 2) a target of MAP2K7 (JNK3A1) that activates complexes involved in transcriptional regulation of stress related genes influencing cell proliferation, apoptosis, motility, metabolism and DNA repair; and 3) NR2C2, a transcription factor activated by JNK1A1 (a drugable molecule influencing retinal cell viability in model systems). We also observed AAMD-related sequence variants resident in genes encoding PPP3CA (a drugable molecule that inactivates MAP3K5), and two genes (TGFB2, TGFBR2) encoding factors involved in MAPK sensing of growth factors/cytokines.

**Conclusions:**

Linkage disequilibrium (LD)-independent genomic enrichment analysis yielded associations of AAMD with aggregates of functionally related genes encoding constituents of the JNK MAPK signaling pathway. FDA-approved drugs now exist to target constituents of stress-activated MAPK pathways and may offer reasonable approaches to preventing or treating AAMD.

## Introduction

More than two million U.S. residents are living with advanced age-related macular degeneration (AAMD) [1], the most common cause of vision loss in people of western European ancestry. [2] The worldwide cost of visual impairment attributable to AMD was estimated at ∼343 billion U.S. dollars ($255 billion in direct health care costs) in a 2010 report by AMD Alliance International. [3] The cardinal lesions of AAMD are proliferative growth of and exudation from outer retinal vessels in the choriocapillaris (*viz*. neovascular AMD) and neurodegeneration of the retinal pigment epithelial (RPE) cell-photoreceptor complex (*viz*. geographic atrophy).[4]–[6] AMD is a common complex disease and its polygenic nature has been partially characterized from 19 strongly-associated (*P*≤1×10^−8^) loci in a multi-center study on over 77,000 people. [7] Ward & Kellis [8] review works demonstrating the influence of common alleles contributing small, but meaningful effects in traits or disease risk [9] – as in the present work, loci associated with complex traits at *P*-values far below those considered significant on a genome-wide level have clustered across the genome within DNA sequence encoding constituents of biological pathways implicated in disease pathogenesis. [10] The central premise here is that inference based on single loci may not elucidate important contributions of genetic variants both manifesting weak associations and the capacity to act together in alteration of disease risk. [11], [12].

Findings from model systems investigating AMD-related pathophysiology [13]–[20] and our studies on the molecular genetics of AMD [21], [22] led us to examine the putative role of stress-activated protein kinase (SAPK) signaling pathways in AMD pathogenesis. Specifically, *in vivo* models of pathologic retinal angiogenesis [23] and age-related macular degeneration [17] have demonstrated that inhibition of c-Jun N-terminal kinase 1 (JNK1), a SAPK, reduces: 1) retinal capillary dropout and neovascular tufting in response to oxidative stress [14]; and 2) apoptosis, vascular endothelial growth factor (VEGF, a key molecule driving retinal angiogenesis) expression, and choroidal neovascularization in response to laser-induced retinal damage. [17].

SAPK cascades contain MAPKs. MAPKs are evolutionarily conserved kinases that act in phospho-relay systems to covalently add phosphate to the side chain of either serine or threonine residues within specific intracellular proteins – the effect of this process is to alter enzymatic activity, catabolism, cellular localization, and interaction of these phosphorylated proteins with other molecules (e.g. protein kinases, phospholipases, transcription factors, and cytoskeletal proteins). [24] MAPK protein phosphatases act to dephosphorylate serine and threonine residues on MAPKs – causing a reversion of MAPKs to inactive states. MAPKs operate in a number of signal transduction networks responsible for cellular sensing and response during gene expression, cell differentiation, movement, division, and death. SAPKs characterize the JNK MAPK and p38 MAPK signaling cascades – JNKs are environmental stress-, radiation-, and growth factor-activated DNA binding proteins that increase activities of the transcription complexes involved in cellular degeneration and pathologic angiogenesis. [14] This body of in vivo evidence guided our focus on stress-activated MAPK cascades.

We have previously reported single locus associations for AAMD with DNA sequence variants resident in genes encoding constituents of MAPK pathways. [21], [22] AAMD-associated single nucleotide polymorphisms (SNPs) also exist in genes encoding cell surface and nuclear receptors known to act in MAPK pathways – such genes include the members of the: 1) peroxisome proliferator activator receptor (PPAR)/retinoid X receptor (RXR) complex [22]; 2) VEGF signaling system[22], [25]–[27]; and, 3) insulin signaling system. [28].

The primary aim of the present study was to determine whether DNA sequence variants in genes encoding constituents of JNK MAPK and p38 MAPK pathways were associated with AAMD. We reasoned that MAPKs and MAPK protein phosphatases of these pathways, their targeting enzymes, ligands, targets, and metabolites may form systems that influence AMD pathogenesis through regulation of *aggregate* transcriptional responses to chronic environmental and biochemical cellular stressors to which the retina is commonly exposed (these include hypoxia, ultraviolet light, inflammatory cytokines [29] and stimuli influencing barrier function [30]).

We applied findings from a genome-wide association project (NEI-AMD, the NEI Study of Age-Related Macular Degeneration) to a pathway-based genome-wide analytic method (INRICH) [11] to test for enriched association signals in five published sets of genes encoding MAPK pathway constituents including SAPKs (see *Methods* section and **Table 1**). In this process we examined linkage disequilibrium (LD)-independent genomic intervals to determine whether common DNA sequence variants within groups of genes encoding constituents of MAPK pathways were, in aggregate, more strongly associated with AAMD than expected by chance alone. This multi-locus method uses positional clustering of SNPs to account for various genomic confounding factors (such as varying gene size, SNP density, LD, and local clustering of functionally related genes) that may otherwise produce spurious increases in the magnitude of association measures favoring risk (the result of which would inflate the false positive rate [31]). AAMD-associated LD-independent genomic intervals containing 1-or-more AAMD-related SNPs were defined from the positional coordinates of sequence variants attaining *P*-values ≤0.002 in meta-analysis on AAMD endpoints from NEI-AMD. NEI-AMD data were from three independent and geographically distinct U.S.-based cohorts of western European ancestry participating in large-scale projects designed to investigate the molecular genetics of AMD (details on study cohorts exist at the National Center for Biotechnology Information, http://www.ncbi.nlm.nih.gov/projects/gap/cgi-bin/study.cgi?study_id=phs000182.v2.p1). The *P*-value threshold of 0.002 for pathway analysis was chosen as it retains about 5% of AAMD-associated genomic loci occurring in genomic regions. We analyzed a total of 1177 people with AAMD and 1024 of their AMD-free peers, aged 65 years-or-older at time of genotyping. SNPs.

**Table 1:** Five published MAPK pathways examined for enrichment with AAMD-associated genomic intervals.

		Genes			Enrichment	*P*-value
Pathway	Source	In Pathway	Tested	AMD-Associated	Empirical	Adjusted
**MAPK** *	BioCarta^a^	86	49	9	0.014	0.037
**MAPK** *	KEGG^b^	261	144	19	0.153	0.339
**JNK MAPK**	STKE^c^	38	25	6	0.002	0.006
**p38 MAPK**	STKE	35	23	5	0.021	0.057
**p38 MAPK**	BioCarta	39	21	5	0.023	0.070

Note: AAMD, advanced age-related macular degeneration;

a information on BioCarta pathways exists at: http://www.biocarta.com;

b information on KEGG pathways exists at: http://www.genome.jp/kegg/pathway (MAPK: hsa04010);

c information on STKE pathways exists at: http://stke.sciencemag.org (JNK MAPK: CMP_10827; p38 MAPK: CMP_10958).

* ‘MAPK’ pathways from BioCarta and KEGG include constituents of the 4 major MAPK cascades (JNK MAPK, p38 MAPK, ERK1/ERK2, ERK5). Empirical *P*-values for pathway interval enrichment were obtained from permutation test applying 100,000 iterations. Enrichment *P*-values were adjusted for multiple testing, as described in the *Methods* section.

We now report linkage disequilibrium-independent genomic enrichment associations of AAMD with aggregates of functionally related genes encoding constituents of stress-activated MAPK signaling pathways. Analysis of AAMD-associated JNK MAPK gene set constituents for co-inheritance with previously identified genome-wide AMD-associated (*P*≤1.0×10^−8^) loci showed null findings (incomplete linkage disequilibrium with r^2^ values <0.20). As multiple drug targets exist in SAPK-resident systems, these may offer reasonable options for preventing or treating AAMD.

## Methods

Data used for genetic analyses in this report were obtained from the NEI-AMD Database at the U.S. National Center for Biotechnology Information (NCBI) database of Genotypes and Phenotypes (dbGaP). Details on NEI-AMD exist at http://www.ncbi.nlm.nih.gov/projects/gap/cgi-bin/study.cgi?study_id=phs000182.v2.p1 (Accessed 2013 July 9). NEI-AMD is a collaborative of researchers from the University of Michigan, Mayo Clinic, University of Pennsylvania, and the Age-Related Eye Disease Study (AREDS) group. NEI-AMD researchers collected clinical data and DNA from people affected with AMD and their elderly AMD-free peers. The NEI was the primary sponsor of this effort. Institutional review boards at each NEI-AMD study site reviewed and approved the study protocols. Each participant provided written informed consent in accordance with the *Declaration of Helsinki*.

### Subjects and Study Design

Details on the NEI-AMD genome-wide association (GWA) study exist at the link in the first paragraph of this section. In brief, three independent cohorts from the University of Michigan in Ann Arbor, the University of Pennsylvania in Philadelphia, and the Mayo Clinic in Rochester, Minnesota contributed data to this genome-wide association study. Our analytic sample contained respectively 675, 227, and 275 people with advanced AMD from the University of Michigan, University of Pennsylvania, and The Mayo Clinic. There were 512, 198, and 314 AMD-free people, aged ≥65 years in these respective sites. We restricted the AMD-free comparison cohort to people ≥65 years-of-age. The purpose of selecting our oldest AMD-free participants was to reduce the chances of including false negatives in analyses.

### Outcome Ascertainment

People with AAMD had GA and/or NV AMD. Experienced graders (ophthalmologists) classified outcomes according to AMD diagnosis in the worse eye. Our AMD-free comparison group had no large or intermediate drusen in either eye, with no more than 5 hard drusen or small drusen and pigment changes in one eye only. If small drusen or pigment changes were present in the AMD-free group, they were neither bilateral nor extensive. All participants received examinations and gradings by the study ophthalmologists. No participant exhibited history or evidence of: 1) retinal insult rendering the fundus ungradable; 2) severe macular disease or vision loss onset prior to 40-years-of-age; or 3) diagnosis of juvenile macular or retinal degeneration, macular damage resulting from ocular trauma, retinal detachment, high myopia, chorioretinal infection, or inflammatory disease, or choroidal dystrophy.

### Genotyping

All specimens were genotyped with DNA microarrays at the Johns Hopkins University Center for Inherited Disease Research (CIDR, Baltimore, MD, USA) using the ILLUMINA HumanCNV370v1 chip. This chip was selected by the NEI Study of Age-Related Macular Degeneration researchers (see link above). The HumanCNV370v1 permits tests on 370,404 DNA sequence variants. The feature set may be accessed on the dbSNP short variations database located at: (http://www.ncbi.nlm.nih.gov/SNP/snp_viewBatch.cgi?sbid=1047132).

### Statistical Analyses

All sequence variants analyzed for AAMD relationships passed process quality and analytic filters for missingness (<5%), minor allele frequency (>1%), and Hardy-Weinberg equilibrium (HWE *P*≤1×10^−6^ in the AMD-free group). We used PLINK (version 1.07, http://pngu.mgh.harvard.edu/purcell/plink/) [32] and SAS (version 9.1, Cary, NC) software for these purposes.

We first examined the allelic distributions of SNPs in people with AMD (relative to the AMD-free comparison group) using age-, sex-, and smoking-adjusted logistic regression analyses. Combined estimates of association were computed using PLINK with meta-analytic techniques under constraints of sample heterogeneity (using Cochrane’s Q for evaluation). *P*-values and odds ratios were chosen from random effects models in instances when significant heterogeneity was demonstrated.

We then used INRICH [11] to examine statistical enrichment of five MAPK pathway gene sets. The five SAPK-containing gene sets were curated by experts in the MAPK signaling field; these were: 1) a general MAPK gene set including constituents of the 4 major MAPK cascades (JNK MAPK, p38 MAPK, ERK1/ERK2, ERK5– containing 86 genes with 49 present on our chip) published at BioCarta (http://www.biocarta.com); 2) a general MAPK gene set (261 genes, 144 present on our chip) published at the Kyoto Encyclopedia of Genes and Genomes pathway maps database (http://www.genome.jp/kegg); 3) a p38 MAPK gene set (35 genes, 23 present on our chip) published by the AAAS Science Signaling Signal Transduction Knowledge Environment (http://stke.sciencemag.org/cm); 4) a p38 MAPK gene set (35 genes, 23 present on our chip) published by BioCarta (39 genes, 21 present on our chip); and, 5) a JNK MAP gene set (38 genes, 25 represented on our chip) published by the AAAS Science Signaling Signal Transduction Knowledge Environment. We examined the general sets to determine whether shared or distinct patterns of association clustered between or within specific MAPK cascades.

AMD-associated LD-independent genomic intervals were computed using tag SNP selection tools in PLINK (r^2^ = 0.50, associated SNPs ≤1000 kb of tag SNP) on variants attaining statistical significance for AMD relationships at *P*≤0.002. This significance threshold was chosen following to previous work [33] that retains the top 5% of genic intervals in the single-variant association tests. We applied 100,000 permutations in our first phase of INRICH enrichment analysis and 10,000 in the bootstrap replication phase for correction of multiple testing.

### Functional Annotation of AAMD-associated MAPK Genes

Information on functional annotation and regulatory features was obtained from HaploReg (www.broadinstitute.org/mammals/haploreg/).

## Results

### MAPK Pathway Enrichment

**Table S1** contains demographic characteristics of our analytic groups. There were 21 genes from the 5 tested MAPK pathway gene sets containing at least one AAMD-associated interval significant at *P*≤0.002. AAMD-associated genomic regions were significantly enriched for genes encoding constituents of four-of-five MAPK pathway gene sets (**Table 1**).

Findings persisted in two gene sets after adjustment for multiple testing. Strongest enrichment signals existed for constituents of the JNK MAPK pathway of 38 genes published in the AAAS *Science Signaling* Signal Transduction Knowledge Environment (STKE, http://stke.sciencemag.org/cgi/cm/stkecmCMP_10827 ). In this gene set, 7 LD-independent AAMD-associated genomic regions existed in 6 genes of 25 tested. The enrichment of the JNK MAPK pathway set was consistently observed when potential regulatory regions (i.e. 20–50 kb up/downstream of transcription starting/ending sites of reference genes) were included as genic regions (adjusted enrichment *P*-value = 0.0062). Both the STKE and BioCarta p38 MAPK pathway gene sets showed marginal significance after accounting for the number of statistical tests performed (the respective adjusted enrichment *P*-values are 0.057 and 0.070).

**Figure 1:**
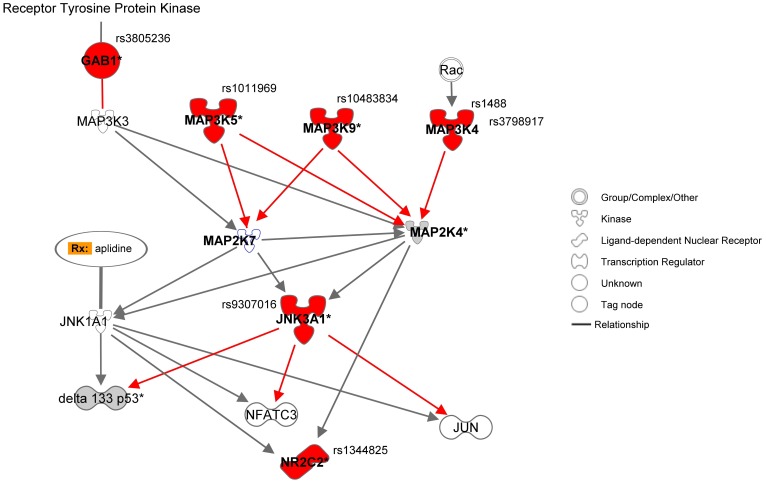
AAMD-associated genes enriching STKE JNK MAPK pathway. Diagram was generated with Ingenuity Pathway Analysis® software and is based on Johnson GL, Lapadat R, JNK Pathway, *Science's STKE*, CMP_10827. Unshaded (white) symbols represent genes that were not tested. Symbols shaded in gray represent relationships with *P*-values >0.002. Symbols shaded in red represent relationships significant at *P*-values ≤0.002. Full names for symbols representing genes exist at www.ncbi.nlm.nih.gov/gene/. Values beneath symbols are *P*-values for association computed with meta-analysis on of age-, sex, and smoking-adjusted odds ratios from 3 independent cohorts participating in large-scale genotyping projects on the molecular genetics of AMD (1177 people with AAMD and 1024 of their AMD-free peers).

**Figure 1** highlights findings on regulators and targets of AAMD-associated constituents within the STKE JNK MAPK Pathway. **Table 2** contains findings for specific SNPs tagging the AAMD-associated intervals in the JNK MAPK pathway set. Most associations showed ∼30% difference in the likelihood of having AAMD associated with the respective MAPK-related sequence variants, after adjusting for age-, sex-, and smoking. **Table 3** contains annotations of all SNPs significant in our analyses. For the STKE JNK MAPK pathway genes, SNPs were present in highly conserved regulatory elements in: 1) three MAP kinase kinase kinases (*MAP3K4*, rs1488, *P*≤6.47×10^−4^ and rs3798917, *P*≤6.87×10^−4^ | *MAP3K5*, rs1011969, *P*≤2.15×10^−3^ | *MAP3K9*, rs10483834, *P*≤1.90×10^−3^) that phosphorylate and activate the MAP kinase kinases MAP2K4 and MAP2K7– these MAP2Ks phosphorylate key threonine and tyrosine residues within the activation loop of JNK; 2) a target of MAP2K7 (*JNK3A1*, rs9307016, *P*≤2.02×10^−3^) that activates complexes involved in transcriptional regulation of stress related genes influencing cell proliferation, apoptosis, motility, metabolism and DNA repair; and 3) a transcription factor activated by JNK1A1 (*NR2C2*, rs1344825, *P*≤2.05×10^−3^) – JNK1A1 (untested in our study) is a drugable DNA binding protein demonstrated to influence retinal vascular cell viability in *in vivo* model systems. [14], [17] AAMD-related sequence variants were also resident in genes encoding PPP3CA (*PPP3CA*, rs2732514, *P*≤5.57×10^−3^) – a drugable molecule that inactivates MAP3K5– and at least two genes (*TGFB2,* rs2796813, ≤1.60×10^−3^; *TGFBR2*, rs1155708, *P*≤3.06×10^−3^) encoding factors involved in JNK MAPK sensing of growth factors/cytokines. **Figure S1** contains information on the location of AAMD-associated genes within the full STKE JNK MAPK Pathway. **Table S4** shows representation of AMD-associated genes by each of the five MAPK gene sets containing SAPKs.

**Table 2:** AAMD-associated sequence variants (*P*≤0.005)* resident in genes of the STKE JNK MAPK pathway.

	SNP Feature		OR (95%CI)				
Symbol	SNP Ref.	Alleles	Cohort 1	Cohort 2	Cohort 3	OR_meta_	*P*_meta_
**GAB1**	rs3805236	T|C	0.68(0.53,0.87)	0.68(0.46,1.00)	0.94(0.66,1.32)	0.74	1.01×10^−3^
**MAP3K4**	rs1488	G|A	1.48(1.16,1.90)	1.58(1.07,2.36)	1.04(0.72,1.49)	1.37	6.47×10^−4^
	rs3798917	G|T	1.45(1.10,1.90)	1.29(0.84,1.98)	1.44(0.97,2.12)	1.41	6.87×10^−4^
**MAP3K5**	rs1011969	A|C	1.30(1.00,1.70)	1.01(0.65,1.57)	1.97(1.31,2.94)	1.37	2.15×10^−3^
	rs9402839	A|G	0.72(0.54,0.95)	0.72(0.46,1.14)	0.78(0.51,1.22)	0.73	4.29×10^−3^
**MAP3K9**	rs10483834	G|A	0.80(0.63,1.03)	0.69(0.47,1.03)	0.69(0.48,0.99)	0.75	1.90×10^−3^
**JNK3A1**	rs9307016	C|T	0.76(0.59,0.99)	0.72(0.47,1.09)	0.69(0.46,1.03)	0.74	2.02×10^−3^
	rs7440491	C|A	0.77(0.60,0.98)	0.84(0.57,1,25)	0.71(0.49,1.04)	0.77	4.81×10^−3^
	rs1469869	T|C	1.29(0.99,1.62)	1.46(0.96,2.20)	1.35(0.91,1.99)	1.34	3.02×10^−3^
**NR2C2**	rs1344825	C|T	0.84(0.66,1.06)	0.54(0.35,0.83)	0.74(0.52,1.09)	0.75	2.05×10^−3^

Note: Odds ratios (ORs) and 95% confidence intervals (95% CIs) were computed, respectively, in Cohort 1/2/3 with age-, sex-, and smoking-adjusted logistic regression models comparing 675/227/275 people with advanced AMD (AAMD) to 512/198/314 of their AMD-free peers, aged >65 years at the time of genotyping. Participants from Cohort 1 were examined at the University of Michigan Ann Arbor. Those in Cohort 2 were examined at the University of Pennsylvania in Philadelphia. Those in Cohort 3 were examined at the Mayo Clinic in Rochester, Minnesota. Alleles are listed as minor|major.

* Only SNPs significant at *P*≤0.002 were considered as AAMD-related in the pathway analysis. In most cases dominant models of inheritance (grouping minor allele homozygotes with heterozygotes) yielded strongest relationships – ORs reported here are from analyses using this model, with the exception of that for *NR2C2* (here the additive model was used). Table S3 contains allelic frequencies of variants in this table.

**Table 3:** Annotations of AAMD-associated sequence variants resident in MAPK pathways.

	SNP Feature			
Symbol^Pathway^	SNP Reference	Location	Regulatory Element	Conserved
**AKT3** a	rs12031994	Intron	Enhancer*, DNase*	No
**CACNA1A** a	rs4926242	Intron		No
**CREB5** d	rs10233653	Intron	Enhancer*, DNase	No
	rs160342	Intron	–	No
	rs160343	Intron	DNase*	Yes*
	rs41276	Intron	–	Yes
**FGF14** a	rs2984842	Intron	–	No
**GAB1** c	**rs3805236**	**Intron**	**–**	**No**
**MAP2K2** a,b	rs350818	3′ UTR*	DNase	No
**MAP3K4** a,b,c	**rs1488**	3′UTR	–	Yes
	**rs3798917**	Intron	ENH*	Yes*
**MAP3K5** a,b,c,d,e	**rs1011969**	**Int., SYN** *	**ENH, DNase** *	**Yes** *
**MAP3K9** b,c,e	**rs10483834**	**Intron**	**PRO** * **, ENH** * **, DNase** *	**Yes** *
**JNK3A1** a,b,c	**rs9307016**	**Intron**	**DNase** *	**Yes** *
**MAPK8IP2** a	rs2238834	Intron	PRO, DNase	No
**MAPKAPK2** a,e	rs4256810	Intron	Enhancer, DNase	Yes*
**NFATC2** a	rs2273642	Intron	DNase*	No
**NR2C2** c	**rs1344825**	**Int., 5′UTR** *	**PRO** * **, ENH** * **, DNase**	**No**
**PLA2G12A** a	rs2285714	SYN, 5′UTR*	PRO*, DNase	Yes
**PLA2G4A** a	rs7519192	Intron	ENH*, DNase (RPE)	Yes*
**PPP3CA** a	rs3804357	Intron	–	No
**RPS6KA2** a,b	rs2072641	Intron	DNase	No
**TAOK3** a	rs514826	Int., 3′UTR*	PRO, DNase	No
	rs571113	Intron	PRO, ENH, DNase*	No
**TGFB2** a,b,e	rs2796813	Intron	PRO*, ENH*, DNase	Yes*
**TGFBR2** a	rs1155708	Intron	PRO*, ENH*, DNase*	Yes*

Note: DNase, DNase hypersensitivity cluster; ENH, histone enhancer mark; Int., variant resident in intron; JNK, genes present in STKE JNK MAPK Pathway (see Tables 1 and S4); PRO, histone promoter mark; RPE, retinal pigment epithelial cell; SNP, single nucleotide polymorphism; SYN, synonymous coding variant; UTR, untranslated region.

* Annotations marked with an asterisk are for qualities of sequence variants in nearly complete linkage disequilibrium (r^2^≥0.80) with the AAMD-associated variant in the SNP column. Full names of genes represented by gene symbols exist at http://www.ncbi.nlm.nih.gov/gene. Information on regulatory features was obtained from HaploReg (www.broadinstitute.org/mammals/haploreg/). Pathway:

a KEGG MAPK Signaling Pathway;

b BioCarta MAPK Signaling Pathway;

c STKE JNK MAPK Pathway;

d STKE p38 MAPK;

e BioCarta p38 MAPK Pathway.

#### Potential Influence of Other AMD-associated SNPs on MAPK Findings

Approximately 20 loci strongly associated with AMD (*P*-values ≤1×10^−8^) were recently reported in a multi-center study on over 77,000 people [7]. Our findings were concordant for a number of these regions – **Table S2** contains a list of AMD-related SNPs statistically significant in our cohort at *P*≤1.0×10^−8^ in age-, sex, and smoking-adjusted models. Conditioning our single locus analyses on the two variants with strongest associations (rs932275, *HTRA1*, *P*≤3.5×10^−48^; rs1329428 *CFH*, *P*≤3.3×10^−55^) attenuated the magnitude of relationships in our MAPK-related SNPs in Table 2; however, all sequence variants discussed in the *MAPK Pathway Enrichment* section (directly above) remained significant at *P*≤0.05. To examine potential coinheritance of SNPs in Table S2 with AMD-associated SNPs of the JNK MAPK AAMD-associated intervals in Table 2, we computed measures of linkage disequilibrium. In no case was the r^2^ value of a SNP in Table S2 with a SNP in Table 2 greater than 0.2. Our conclusion is that findings for the MAPK-pathway-resident SNPs are biologically plausible and concordant with those in the model systems.

### AAMD-associated SNPs in Genes Encoding Factors Driving JNK MAPK Sensing and Response

*Via* receptor tyrosine kinases (RTKs), cytokine receptors, AND guanine nucleotide (G)-protein coupled receptors (GPCRs) JNK MAPK cascade constituents sense and respond to hypoxia, radiation, survival and growth factors, cytokines, chemokines, hormones, and amino acid neurotransmitters. We extended our examination of the JNK MAPK cascade components to include DNA variants in SAPK system genes encoding ligands, receptors, and molecules embedded in, or associated with the plasma membrane dynamics.

#### Survival Factors

Cell survival factors activate SAPK-driven signal transduction pathways *via* RTKs. VEGF is a key pro-angiogenic factor in vasoproliferative retinopathies in its capacity to induce endothelial cell migration and proliferation, microvascular permeability, endothelial cell release of metalloproteinases and interstitial collagenases, and endothelial cell tube formation.[34]–[36] VEGF activates MAPKs *via* insulin-like growth factor 1 (IGF1) and recent work on primary human RPE (hRPE) cells has demonstrated that constituents of MAPK pathways are likely to influence IGF1-mediated retinal angiogenesis. [15] We observed AAMD associations with a number of SNPs in insulin signaling system genes. Most notable was a missense variant in insulin-like growth factor-binding protein 1 (*IGFBP1*, Ile253Met, rs4619, *P*≤0.003) that yields an amino acid substitution proximal to a cell attachment site in the mature protein (NCBI Reference Sequence: NP_000587.1). We also observed relationships of AMD with a highly conserved 3′ UTR-resident SNP in *IGF1* (rs6219, *P*≤0.015) and a highly conserved intronic variant overlapping histone promoter marks in insulin-like growth factor-binding protein 5 (*IGFBP5*, rs3770472, *P*≤0.006).

#### Growth Factors

Growth factors activate SAPK-driven signal transduction pathways *via* RTKs. We have discussed our findings for *TGBFB2* and *TGFBR2*, above. Activation of the JNK MAPK cascade by growth factors leads, through the small GTPase Ras, to the activation of 1-phosphatidylinositol 3-kinase (PI3K) complexes. We observed AAMD-associated sequence variants in genes encoding regulatory subunit 4 phosphatidylinositol 3-kinase (*PIK3R4*, rs11713445, *P*≤1.37×10^−4^ | rs2200368, *P*≤2.11×10^−4^) and the 3′ untranslated region (UTR) of regulatory subunit 6 phosphatidylinositol 3-kinase (*PIK3R6*, rs2242375, *P*≤1.81×10^−3^). Growth factor-induced JNK MAPK cascade activation may also proceed post-binding at the RTK *via* an interaction of growth factor receptor bound protein 2 (GRB2) with GRB2-associated binding protein 1 (GAB1). We observed an AAMD-associated SNP in *GAB1* (rs3805236, *P*≤1.01×10^−3^).

#### Chemokines, Hormones, Amino Acid Transmitters

Chemokines, hormones, and amino acid transmitters activate SAPK-driven signal transduction pathways *via* GPCRs. After ligands bind GPCRs at the plasma membrane, G-protein beta and gamma binding proteins are activated and increase activation of phosphatidylinositol 3-kinase gamma in a cascade subsequently involving Rac and MAP3K9. We observed AAMD-associated variants in an intronic area with histone promoter marks in G-protein beta polypeptide 3 (*GNB3*, rs4334421, *P*≤2.20×10^−3^) and a highly conserved missense variant in G-protein gamma 7 (*GNG7*, rs5442, Gly272Ser, *P*≤1.03×10^−3^) that yields a residue change within an evolutionarily conserved aspartate-tryptophan repeat sequence (WD 1) partially responsible for forming the 3-D structure of the mature protein.

## Discussion

Using data from a whole genome genotyping microarray and a multi-locus enrichment analysis [11], we have identified AAMD-associated regions related to a ∼30% change in the likelihood of having AAMD within 6-of-25 tested constituents of the stress-activated protein JNK MAPK signaling cascade (a pathway involved in cellular sensing and response during gene expression, cell differentiation, movement, division, and death); AAMD-associated SNPs resident in MAPK pathway genes were not co-inherited with previously identified high risk loci. In addition to identifying AAMD-associated factors that may act in aggregate response to stress from processes implicated in AMD pathogenesis, we now report on a number of AAMD-associated factors involved as constituents of sensing modules essential to MAPK response complex activation.

While the magnitude of relationships from our pathway-based analyses was less than those conventionally reported for single locus tests of genome wide significance, our findings offer interpretation with strong biological plausibility. JNK and p38 mitogen activated protein kinases (MAPKs) have been linked in a cell system to mRNA stability of VEGF. Pagès *et al*. used a Chinese hamster lung fibroblast line (CCL39) to demonstrate that non-specific pharmacologic activation of JNKs altered post-transcriptional regulation of VEGF by increasing VEGF mRNA stabilization. [13] The same team reported that VEGF mRNA induction is inhibited by JNK blockade with the drug SB202190. Guma *et al*. [14] used an *in vivo* murine model of pathologic retinal angiogenesis [23] to demonstrate that JNK1 is a critical factor in hypoxia-induced retinal VEGF production; this team provided the first evidence that genetic JNK1 deficiency (JNK1^−/−^ mouse) and pharmacologic JNK1 inhibition (D-JNKi) ameliorates retinal capillary dropout and neovascular tufting in response to oxidative stress. Du *et al*. used a murine model of neovascular AMD to demonstrate that JNK1 deficiency or JNK inhibition leads to a decrease in apoptosis, VEGF expression, and reduction of choroidal neovascularization. The choroid is the primary vascular bed damaged in AMD. [17] A number of model cell systems in immortalized RPE cells have implicated constituents of the JNK MAPK pathway in retinal cell survival.

The BioCarta® MAPK Pathway gene set (http://www.biocarta.com) also showed significant enrichment of AMD-associated genes (adjusted *P*-value = 0.037). This pathway gene set contains constituents of the SAPK cascades (JNK MAPK and p38 MAPK), the extra-cellular-regulated kinases (ERK1 and ERK2, mitogen-activated MAPKs), and the ERK5 cascade (a system of stress- and mitogen-activated extra-cellular-regulated kinases). Although there was no clear evidence in the enrichment of AAMD-associated genes within a specific constituent, we see a reasonable basis to examine mitogen-activated (ERK1/ERK2) cascades in future studies. As discussed above, VEGF activates MAPKs *via* insulin-like growth factor (IGF1). PD98059, a selective inhibitor of MAPK kinases ERK1 and ERK2, suppressed IGF1-induced hRPE cell proliferation and inhibited IGF1-induced VEGF synthesis. [15] Anathrax lethal toxin (LeTx) has a similar activity profile to PD98059 in cancer cell lines [37] and is known to inactivate MAPK signaling pathways by cleaving the amino termini of MAP2K1, MAP2K2, MAP2K3, MAP2K4, MAP2K6, and MAP2K7 (reviewed in ref [16]). Bromberg-White *et al*. [16] used an *in vivo* murine model of retinal vascular development to examine the effects LeTx on nascent retinal vascular networks. Injecting LeTx during a critical developmental phase characterized by the presence of active MAPK in sprouting (nascent) retinal capillaries led to abrogation of branching morphogenesis in the inner retinal plexus. Four days post-injection, florid focal retinal neovascular growth occurred in the outer retina; this pathologic condition was linked to elevated vitreal VEGF concentrations.

Our inferences were constrained by the coverage of sequence variation on the genotyping platform, natural history (late-onset presentation) of AMD, and a priori-selection of a significance threshold. It is important to acknowledge that our data on genotypes are from a convenience sample of loci available on the commercial (*viz.* non-customized) 300 K micro-arrays we used. The power of this study is thus limited by the sparse coverage of the genotyping chip. In no instance did we have data on all genes in a pathway – and the data available were not collected with the intention of examining specific protein coding regions or areas overlapping active regulatory sites. Regarding the AMD endpoint: because the likelihood of having AMD increases 2-to-6 fold after age 75, we conducted careful selection of an appropriate elderly AMD-free comparison group (≥65-years-of-age) that was chosen to reduce the likelihood of misclassifying people in subclinical phases of AMD as controls. Lastly, overrepresentation-based enrichment methods, such as INRICH [11] require a pre-specified *P*-value threshold to define a list of significant SNP/gene/intervals as input. Clearly, this nominal *P*-value threshold is critical in determining the power of the study, however, the optimal value would largely depend on the proportion of truly trait-associated GWAS signals vs. false-positive ones, which is unknown *a-priori* and specific to each phenotype [38]. As such, although we confirmed a significant enrichment of pathway genes using the `pathway enrichment global p-value *G_p_*’ output (*G_p_*<0.006) [11] using the top 5% of association signals, we could have missed stronger enrichment of the MAPK pathway genes tested under different *P*-value thresholds.

In summary, we have provided novel findings on the relationship of MAPK pathway constituents with AAMD. Common DNA sequence variants within groups of genes encoding both response and sensing constituents of these pathways were, in aggregate, more strongly associated with AAMD than expected by chance alone. FDA-approved drugs now exist to target constituents of these pathways and may offer reasonable approaches to preventing or treating AAMD.

## Supporting Information

Figure S1 **Distribution of AAMD-associated genes enriching STKE JNK MAPK pathway.** Diagram was generated with Ingenuity Pathway Analysis® software and is based on Johnson GL, and Lapadat R, JNK Pathway, *Science’s STKE*, CMP_10827. Unshaded (white) symbols represent genes that were not tested. Symbols shaded in gray represent relationships with *P*-values >0.005. Symbols shaded in red represent relationships significant at *P*-values ≤0.005. Full names for symbols representing genes exist at www.ncbi.nlm.nih.gov/gene/. Values beneath symbols are *P*-values for association computed with meta-analysis on of age-, sex, and smoking-adjusted odds ratios from 3 independent cohorts participating in large-scale genotyping projects on the molecular genetics of AMD (1177 people with AAMD and 1024 of their AMD-free peers).
(TIF)

Table S1 **Description of Cohorts.**
(DOCX)

Table S2 **SNPs associated with advanced AMD at** ***P*** **≤1×10^−8^ in age-, sex-, and smoking-adjusted logistic regression models.**
(DOCX)

Table S3 **Allele frequencies for AAMD-associated JNK MAPK pathway gene set variants.**
(DOCX)

Table S4 **Genes with AAMD-associated sequence variants within MAPK pathways.**
(DOCX)
