# Supplementary figures and images for: AMD-Associated Genes Encoding Stress-Activated MAPK Pathway Constituents Are Identified by Interval-Based Enrichment Analysis
Source: PLoS One. 2013 Aug 5;8(8):e71239. doi: 10.1371/journal.pone.0071239 (PMC3734129; doi:10.1371/journal.pone.0071239)

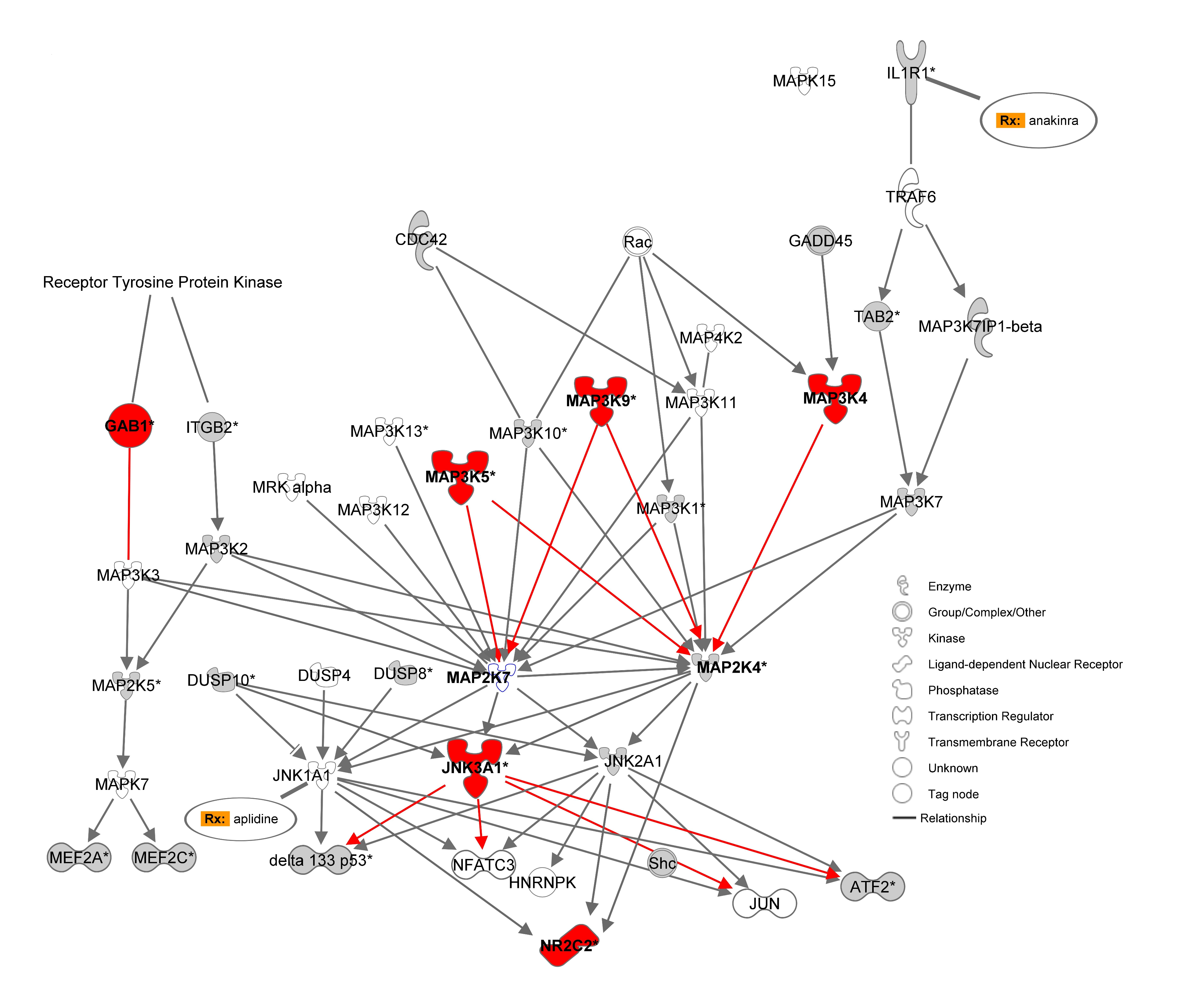

Supplement: Figure S1 — Distribution of AAMD-associated genes enriching STKE JNK MAPK pathway. Diagram was generated with Ingenuity Pathway Analysis® software and is based on Johnson GL, and Lapadat R, JNK Pathway, Science’s STKE, CMP_10827. Unshaded (white) symbols represent genes that were not tested. Symbols shaded in gray represent relationships with P-values >0.005. Symbols shaded in red represent relationships significant at P-values ≤0.005. Full names for symbols representing genes exist at www.ncbi.nlm.nih.gov/gene/. Values beneath symbols are P-values for association computed with meta-analysis on of age-, sex, and smoking-adjusted odds ratios from 3 independent cohorts participating in large-scale genotyping projects on the molecular genetics of AMD (1177 people with AAMD and 1024 of their AMD-free peers). (TIF) [file pone.0071239.s001.tif]
