# Supplementary material for: AMD-Associated Genes Encoding Stress-Activated MAPK Pathway Constituents Are Identified by Interval-Based Enrichment Analysis
Source: PLoS One. 2013 Aug 5;8(8):e71239. doi: 10.1371/journal.pone.0071239 (PMC3734129; doi:10.1371/journal.pone.0071239)
Supplement: Table S1 — Description of Cohorts. (DOCX) [file pone.0071239.s002.docx]

Table S1. Description of Cohorts.

|  |  | **Outcome** |  |  |
| --- | --- | --- | --- | --- |
| **Cohort** |  | **No AMD** |  | **AAMD** |
| Cohort 1^a^ |  |  |  |  |
| Total, N |  | 514 |  | 675 |
| Mean age at exam (SE) |  | 76.6 (0.23) |  | 80.6 (0.27) |
| Female (%) |  | 58 |  | 63 |
| Current smoker (%) |  | 4 |  | 7 |
| Cohort 2^b^ |  |  |  |  |
| Total, N |  | 198 |  | 227 |
| Mean age at exam (SE) |  | 76.2 (0.34) |  | 76.8 (0.46) |
| Female (%) |  | 55 |  | 60 |
| Current smoker (%) |  | 7 |  | 6 |
| Cohort 3^c^ |  |  |  |  |
| Total, N |  | 318 |  | 275 |
| Mean age at exam (SE) |  | 73.7(0.34) |  | 79.7 (0.44) |
| Female (%) |  | 53 |  | 62 |
| Current smoker (%) |  | 6 |  | 7 |

Note: a = University of Michigan, b = University of Pennsylvania, c = Mayo Clinic, Rochester SE, standard error; AAMD, advanced AMD.
