# Supplementary material for: AMD-Associated Genes Encoding Stress-Activated MAPK Pathway Constituents Are Identified by Interval-Based Enrichment Analysis
Source: PLoS One. 2013 Aug 5;8(8):e71239. doi: 10.1371/journal.pone.0071239 (PMC3734129; doi:10.1371/journal.pone.0071239)
Supplement: Table S2 — SNPs associated with advanced AMD at P ≤1×10^−8^ in age-, sex-, and smoking-adjusted logistic regression models. (DOCX) [file pone.0071239.s003.docx]

Table S2. SNPs associated with advanced AMD at *P* < 1 x 10^-8^  in age-, sex-, and smoking-adjusted logistic regression models.

| *P*-value | SNP |  | Symbol | Gene Name |
| --- | --- | --- | --- | --- |
| 3.29E-55 | rs1329428 |  | CFH | complement factor H |
| 3.53E-48 | rs932275 |  | HTRA1 | HtrA serine peptidase 1 |
| 5.61E-45 | rs2019724 |  | CFH | complement factor H |
| 5.70E-39 | rs6667243 |  |  |  |
| 2.52E-35 | rs10801555 |  | CFH | complement factor H |
| 2.79E-35 | rs10754199 |  | CFH | complement factor H |
| 7.43E-29 | rs800292 |  | CFH | complement factor H |
| 2.13E-28 | rs6685931 |  | CFHR4 | complement factor H-related 4 |
| 1.09E-27 | rs505102 |  | CFH | complement factor H |
| 9.13E-27 | rs6677604 |  | CFH | complement factor H |
| 5.52E-25 | rs2284664 |  | CFH | complement factor H |
| 1.31E-24 | rs2248799 |  | HTRA1 | HtrA serine peptidase 1 |
| 2.24E-23 | rs4915318 |  |  |  |
| 7.57E-23 | rs6657442 |  |  |  |
| 3.24E-22 | rs10922144 |  |  |  |
| 4.50E-22 | rs1332666 |  |  |  |
| 3.53E-19 | rs10754196 |  | KCNT2 | potassium channel, subfamily T, member 2 |
| 7.90E-18 | rs3927686 |  | KCNT2 | potassium channel, subfamily T, member 2 |
| 1.13E-17 | rs3766404 |  | CFH | complement factor H |
| 1.44E-17 | rs2878557 |  | KCNT2 | potassium channel, subfamily T, member 2 |
| 1.54E-17 | rs7535696 |  | KCNT2 | potassium channel, subfamily T, member 2 |
| 1.58E-17 | rs7555070 |  |  |  |
| 2.06E-17 | rs2014307 |  |  |  |
| 2.31E-17 | rs2027368 |  | KCNT2 | potassium channel, subfamily T, member 2 |
| 6.72E-17 | rs6585827 |  | PLEKHA1 | pleckstrin homology domain containing, family A (phosphoinositide binding specific) member 1 |
| 1.34E-16 | rs2280141 |  |  |  |
| 2.14E-16 | rs10801575 |  |  |  |
| 5.72E-16 | rs7517126 |  |  |  |
| 1.96E-15 | rs2292627 |  |  |  |
| 2.18E-15 | rs4086175 |  |  |  |
| 2.65E-15 | rs13375144 |  |  |  |
| 2.86E-15 | rs7548070 |  |  |  |
| 5.10E-15 | rs429608 |  | SKIV2L | superkiller viralicidic activity 2-like (S. cerevisiae) |
| 5.28E-15 | rs7538501 |  |  |  |
| 9.86E-14 | rs11200583 |  |  |  |
| 3.30E-13 | rs4751890 |  | PLEKHA1 | pleckstrin homology domain containing, family A (phosphoinositide binding specific) member 1 |
| 3.69E-13 | rs7522952 |  | CFHR4 | complement factor H-related 4 |
| 7.87E-13 | rs1042663 |  | C2 | complement component 2 |
| 9.63E-13 | rs497239 |  | C2 | complement component 2 |
| 1.34E-12 | rs541862 |  | CFB | complement factor B |
| 1.34E-12 | rs547154 |  | C2 | complement component 2 |
| 1.40E-12 | rs438999 |  | SKIV2L | superkiller viralicidic activity 2-like (S. cerevisiae) |
| 1.46E-12 | rs550605 |  | C2 | complement component 2 |
| 1.95E-12 | rs4751889 |  | PLEKHA1 | pleckstrin homology domain containing, family A (phosphoinositide binding specific) member 1 |
| 1.40E-10 | rs7080960 |  |  |  |
| 2.04E-10 | rs10754210 |  | F13B | coagulation factor XIII, B polypeptide |
| 2.34E-10 | rs10494744 |  | KCNT2 | potassium channel, subfamily T, member 2 |
| 5.70E-10 | rs511294 |  | C2 | complement component 2 |
| 6.46E-10 | rs6003 |  | F13B | coagulation factor XIII, B polypeptide |
| 7.02E-10 | rs2026429 |  | ASPM | asp (abnormal spindle) homolog, microcephaly associated (Drosophila) |
| 1.00E-09 | rs544167 |  | C2 | complement component 2 |
| 1.35E-09 | rs10494743 |  | KCNT2 | potassium channel, subfamily T, member 2 |
| 1.65E-09 | rs12034362 |  | ASPM | asp (abnormal spindle) homolog, microcephaly associated (Drosophila) |
| 1.84E-09 | rs10754220 |  | CRB1 | crumbs homolog 1 (Drosophila) |
| 1.86E-09 | rs10801538 |  | KCNT2 | potassium channel, subfamily T, member 2 |
| 2.02E-09 | rs2250656 |  | C3 | complement component 3 |
| 2.39E-09 | rs2300431 |  | HTRA1 | HtrA serine peptidase 1 |
| 3.11E-09 | rs7554267 |  | KCNT2 | potassium channel, subfamily T, member 2 |
| 5.48E-09 | rs12137359 |  | ZBTB41 | zinc finger and BTB domain containing 41 |
| 1.17E-08 | rs10801533 |  | KCNT2 | potassium channel, subfamily T, member 2 |
