# Supplementary material for: AMD-Associated Genes Encoding Stress-Activated MAPK Pathway Constituents Are Identified by Interval-Based Enrichment Analysis
Source: PLoS One. 2013 Aug 5;8(8):e71239. doi: 10.1371/journal.pone.0071239 (PMC3734129; doi:10.1371/journal.pone.0071239)
Supplement: Table S3 — Allele frequencies for AAMD-associated JNK MAPK pathway gene set variants. (DOCX) [file pone.0071239.s004.docx]

Table S3. Allele frequencies for AAMD-associated JNK MAPK pathway gene set variants.

|  |  | Alleles |  |  | AAMD |  |  |  | No AMD |  |  |
| --- | --- | --- | --- | --- | --- | --- | --- | --- | --- | --- | --- |
| Symbol | SNP Ref. | Minor (A) | Major (B) |  | AA | AB | BB |  | AA | AB | BB |
| GAB1 | rs3805236 | T | C |  | 176 | 733 | 807 |  | 128 | 541 | 480 |
| MAP3K4 | rs1488 | G | A |  | 209 | 825 | 670 |  | 143 | 489 | 511 |
| MAP3K4 | rs3798917 | G | T |  | 48 | 510 | 1145 |  | 30 | 276 | 828 |
| MAP3K5 | rs1011969 | A | C |  | 41 | 479 | 1199 |  | 21 | 277 | 852 |
| MAP3K5 | rs9402839 | A | G |  | 34 | 351 | 1333 |  | 18 | 268 | 864 |
| MAP3K9 | rs10483834 | G | A |  | 100 | 594 | 1023 |  | 62 | 448 | 640 |
| JNK3A1 | rs9307016 | C | T |  | 40 | 422 | 1256 |  | 31 | 345 | 774 |
| JNK3A1 | rs7440491 | C | A |  | 84 | 538 | 1096 |  | 62 | 421 | 665 |
| JNK3A1 | rs1469869 | T | C |  | 340 | 838 | 541 |  | 206 | 548 | 396 |
| NR2C2 | rs1344825 | C | T |  | 107 | 699 | 909 |  | 104 | 482 | 561 |
