# Supplementary material for: AMD-Associated Genes Encoding Stress-Activated MAPK Pathway Constituents Are Identified by Interval-Based Enrichment Analysis
Source: PLoS One. 2013 Aug 5;8(8):e71239. doi: 10.1371/journal.pone.0071239 (PMC3734129; doi:10.1371/journal.pone.0071239)
Supplement: Table S4 — Genes with AAMD-associated sequence variants within MAPK pathways. (DOCX) [file pone.0071239.s005.docx]

Table S4. Genes with AAMD-associated sequence variants within MAPK pathways.

|  |  | Pathway |  |  |  |  |  |  |  |  |
| --- | --- | --- | --- | --- | --- | --- | --- | --- | --- | --- |
| Symbol |  | **MAPK^a^** | **MAPK^b^** |  | **JNK MAPK^c^** |  | **p38 MAPK^d^** |  | **p38 MAPK^e^** |  |
| AKT3 |  | ● |  |  |  |  |  |  |  |  |
| CACNA1A |  | ● |  |  |  |  |  |  |  |  |
| CREB5 |  |  |  |  |  |  | ● |  |  |  |
| FGF14 |  | ● |  |  |  |  |  |  |  |  |
| GAB1 |  |  |  |  | ● |  |  |  |  |  |
| MAP2K2 |  | ● | ● |  |  |  |  |  |  |  |
| MAP3K13 |  | ● | ● |  | ● |  |  |  |  |  |
| MAP3K4 |  | ● | ● |  | ● |  | ● |  |  |  |
| MAP3K5 |  | ● | ● |  | ● |  | ● |  | ● |  |
| MAP3K9 |  |  | ● |  | ● |  |  |  | ● |  |
| JNK3A1 |  | ● | ● |  | ● |  |  |  |  |  |
| MAPK8IP2 |  | ● |  |  |  |  |  |  |  |  |
| MAPKAPK2 |  | ● | ● |  |  |  |  |  | ● |  |
| NFATC2 |  | ● |  |  |  |  |  |  |  |  |
| NR2C2 |  |  |  |  | ● |  | ● |  |  |  |
| PLA2G12A |  | ● |  |  |  |  |  |  |  |  |
| PLA2G4A |  | ● |  |  |  |  |  |  | ● |  |
| PPP3CA |  | ● |  |  |  |  |  |  |  |  |
| RPS6KA2 |  | ● | ● |  |  |  |  |  |  |  |
| TAOK3 |  | ● |  |  |  |  |  |  |  |  |
| TGFB2 |  | ● | ● |  |  |  |  |  | ● |  |
| TGFBR2 |  | ● |  |  |  |  |  |  |  |  |

Note: a, KEGG MAPK Signaling Pathway; b, BioCarta MAPK Signaling Pathway; c, STKE JNK MAPK Pathway; d, STKE p38 MAPK; e, BioCarta p38 MAPK Pathway. Table 1 contains references for the pathways represented in this table. Full names of genes represented by gene symbols exist at <http://www.ncbi.nlm.nih.gov/gene>.
